# Dopamine Receptor D5 Signaling Plays a Dual Role in Experimental Autoimmune Encephalomyelitis Potentiating Th17-Mediated Immunity and Favoring Suppressive Activity of Regulatory T-Cells

**DOI:** 10.3389/fncel.2018.00192

**Published:** 2018-07-10

**Authors:** Francisco Osorio-Barrios, Carolina Prado, Francisco Contreras, Rodrigo Pacheco

**Affiliations:** ^1^Laboratorio de Neuroinmunología, Fundación Ciencia & Vida, Santiago, Chile; ^2^Departamento de Ciencias Biológicas, Facultad de Ciencias de la Vida, Universidad Andres Bello, Santiago, Chile

**Keywords:** Th17 response, T-cell activation, regulatory T-cells, dopamine, knockout mice, experimental autoimmune encephalomyelitis, neuroimmunology

## Abstract

A number of studies have shown pharmacologic evidence indicating that stimulation of type I dopamine receptor (DR), favors T-helper-17 (Th17)-mediated immunity involved in experimental autoimmune encephalomyelitis (EAE) and in some other inflammatory disorders. Nevertheless, the lack of drugs that might discriminate between DRD1 and DRD5 has made the pharmacological distinction between the two receptors difficult. We have previously shown genetic evidence demonstrating a relevant role of DRD5-signaling in dendritic cells (DCs) favoring the CD4^+^ T-cell-driven inflammation in EAE. However, the role of DRD5-signaling confined to CD4^+^ T-cells in the development of EAE is still unknown. Here, we analyzed the functional role of DRD5-signaling in CD4^+^ T-cell-mediated responses and its relevance in EAE by using a genetic approach. Our results show that DRD5-signaling confined to naive CD4^+^ T-cells exerts a pro-inflammatory effect promoting the development of EAE with a stronger disease severity. This pro-inflammatory effect observed for DRD5-signaling in naive CD4^+^ T-cells was related with an exacerbated proliferation in response to T-cell activation and to an increased ability to differentiate toward the Th17 inflammatory phenotype. On the other hand, quite unexpected, our results show that DRD5-signaling confined to Tregs strengthens their suppressive activity, thereby dampening the development of EAE manifestation. This anti-inflammatory effect of DRD5-signaling in Tregs was associated with a selective increase in the expression of glucocorticoid-induced tumor necrosis factor receptor-related protein (GITR), which has been described to play a critical role in the expansion of Tregs. Our findings here indicate a complex role for DRD5-signaling in CD4^+^ T-cells-driven responses potentiating early inflammation mediated by effector T-cells in EAE, but exacerbating suppressive activity in Tregs and thereby dampening disease manifestation in late EAE stages.

## Introduction

During last 15 years dopamine has emerged as a major regulator of inflammation. All five dopamine receptors (DRs, DRD1-DRD5) have been found to be expressed in immune cells, including dendritic cells (DCs) and T-cells among others, where they exert a complex regulation of immunity (Pacheco et al., 2014). The outcome of the dopamine effects in the immune response depends in many factors, including differential expression of DRs in the immune cells present in the inflamed tissue, the local levels of dopamine and the signaling coupled to and the affinity of the different DRs involved. Current evidence has indicated that stimulation of low-affinity DRs, for instance DRD1 and DRD2, are coupled to anti-inflammatory mechanisms, thereby dampening inflammation (Shao et al., 2013; Yan et al., 2015). Conversely, signaling triggered by high-affinity DRs, including DRD3 and DRD5, has been found consistently to promote inflammation (Prado et al., 2012, 2018; Contreras et al., 2016).

Multiple sclerosis (MS) is an inflammatory disorder involving a CD4^+^ T-cells driven response against the myelin sheath in the central nervous system (CNS). Previous evidence suggested that T-helper-1 (Th1) as well as T-helper-17 (Th17) cells are the phenotypes of autoreactive T-cells involved in MS and its mouse model, the experimental autoimmune encephalomyelitis (EAE; Dardalhon et al., 2008; Oukka, 2008). Importantly, DCs by producing interleukin 12 (IL-12) and IL-23, might induce the differentiation of naive CD4^+^ T-cells into Th1 and Th17 cells respectively (Haines et al., 2013; O’Connor et al., 2013). Consistent with the prominent role of dopamine in regulating adaptive immunity, relevant effects for dopaminergic-signaling have been found in MS (Prado et al., 2012, 2018). In this regard, CD4^+^ T-cells infiltrate the CNS where they can be exposed to dopamine and other neurotransmitters. In fact, striatal dopamine levels are significantly altered in mouse models of MS (Balkowiec-Iskra et al., 2007). Accordingly, emerging evidence has shown a relevant role of type I DRs (including DRD1 and DRD5) in the regulation of CD4^+^ T-cell-mediated autoimmune response involved in human individuals undergoing MS and in EAE (Prado et al., 2013). In this regard, type I DRs have been described to be expressed in DCs (Nakano et al., 2008; Prado et al., 2012) and CD4^+^ T-cells from human and mouse origin (Kipnis et al., 2004; Cosentino et al., 2007; Kim et al., 2013; Franz et al., 2015).

The first work addressing the role of DRs in T-cell mediated immunity, showed that antagonizing type I DRs expressed on human DCs altered acquisition of functional phenotype by naive CD4^+^ T-cells increasing the Th1-to-Th17 ratio in co-cultures of DCs and T-cells *in vitro* (Nakano et al., 2008). Moreover, the same authors reported later that human DCs contain intracellular vesicles loaded with dopamine, which are released during Ag-presentation to naive CD4^+^ T-cells *in vitro* (Nakano et al., 2009). The *in vivo* relevance of these observations was evaluated by using a pharmacological approach in EAE (Nakano et al., 2008). In that study, the treatment of mice with the systemic administration of a type I DRs antagonist, (*R*)-(+)-7-Chloro-8-hydroxy-3-methyl-1-phenyl-2,3,4,5-tetrahydro-1*H*-3-benzazepine hydrochloride (SCH23390), reduced disease severity by impairing the Th17 response. However, this pharmacologic approach does not allow the discrimination between the effects mediated by DRD5 or DRD1 since the drug inhibits both type I DRs with similar affinities (Bourne, 2001). Furthermore, this study could not identify the cell-type responsible for the anti-inflammatory effect of type I DRs antagonism. Importantly, we have contributed to elucidate this mechanism using a genetic approach that allows us to determine the contribution of DRD5-signaling in DCs and its consequences in EAE development. In agreement with previous studies (Nakano et al., 2008), we demonstrate that DRD5-deficient mice exhibited delayed EAE progression with reduced severity compared with normal mice (Prado et al., 2012). In addition, we demonstrated that DRD5-deficiency confined to DCs resulted in exacerbated activation of signal transducer and activator of transcription 3 (STAT3), triggering impaired production of IL-12 and IL-23 with a consequent attenuation in Th1 and Th17 responses and reduced EAE manifestation (Prado et al., 2018). Nevertheless, the extent of attenuation of disease severity was stronger when EAE manifestation was compared between wild-type (WT) and general DRD5 knockout (DRD5KO) mice than when comparing WT animals with mice harboring DRD5-deficiency confined to DCs, suggesting that DRD5 expressed in other cell types different of DCs were also relevant in the regulation of EAE development (Prado et al., 2012).

Addressing the dopaminergic regulation of CD4^+^ T-cells mediated by type I DRs, pharmacological evidence has suggested that signaling triggered by these receptors in human T-cells *in vitro* favors the differentiation toward the Th2 phenotype (Nakano et al., 2009). Another study performed in a mouse model of ovalbumin (OVA)-induced acute asthma shows that pharmacologic antagonism of type I DRs impaired Th17 function and thereby ameliorated the allergic response (Gong et al., 2013). In addition, our previous results using a genetic approach have shown that DRD5-stimulation in mouse CD4^+^ T-cells favors T-cell activation and without detectable effects in Th1 differentiation when activated with Abs to CD3 and CD28 and a Th1-biased mixture of blocking Abs and cytokine milieu *in vitro* (Franz et al., 2015). Regarding the role of type I DRs on Tregs physiology, two independent groups have shown pharmacological evidence indicating that, by stimulating DRD1/DRD5, dopamine reduces the suppressive function of Tregs (Kipnis et al., 2004; Cosentino et al., 2007). This dopamine-mediated inhibitory mechanism involves a reduction in IL-10 and transforming growth factor β (TGF-β) production and diminished expression of cytotoxic T-lymphocyte antigen 4 (CTLA4), which participate in the cytokine-mediated and contact-mediated suppression exerted by Tregs, respectively. Together, these findings support an important role for type I DRs in the regulation of CD4^+^ T-cells physiology and reveal a relevant involvement of these receptors in autoimmunity. Nonetheless, the precise contribution of DRD1- and DRD5-signaling in the regulation of the CD4^+^ T-cell mediated autoimmune response associated to EAE remains unknown.

In this study, we analyzed the precise role of DRD5-signaling in the CD4^+^ T-cell response *in vivo* using a genetic approach. For this purpose, we dissected the role of DRD5 expressed in naive CD4^+^ T-cells and Tregs from that of DRD5 expressed in other hematopoietic cells in EAE. Afterward, the role of DRD5 expressed in CD4^+^ T-cells in inflammation was validated in other *in vivo* paradigms. Our results indicate that DRD5-signaling in CD4^+^ T-cells favors T-cell activation and contributes significantly to the differentiation toward the Th17-inflammatory phenotype *in vivo*. Furthermore, our data show unexpectedly that DRD5-signaling potentiates the suppressive activity of Tregs *in vivo* and *in vitro*. Thus, our findings indicate that DRD5-signaling in CD4^+^ T-cells plays a dual role in EAE development favoring early to the Th17-mediated inflammation and later contributing to the suppressive activity of Tregs.

## Materials and Methods

### Animals

C57BL/6 WT (*cd45.2*^+/+^) mice and B6.SJL-*Ptprc^a^* (*cd45.1*^+/+^) mice in the C57BL/6 background were purchased from The Jackson Laboratory (Bar Harbor, ME, USA). DRD5KO mice were kindly donated by Dr. David Sibley (Hollon et al., 2002), which were back-crossed for at least ten-generations in the C57BL/6 (*cd45.2*^+/+^) genetic background. OVA-specific OT-II transgenic mice expressing specific T-cell receptors (TCRs) for I-A^b^/OVA_323–339_ in the C57BL/6 (*cd45.2*^+/+^) genetic background were gently donated by Dr. María Rosa Bono (Ureta et al., 2007). DRD5KO/OT-II mice were generated by crossing parental DRD5KO and OT-II mice. Six-to-10 weeks old mice were used in all experiments. All mice were maintained and manipulated according to institutional guidelines at the pathogen-free facility of the Fundación Ciencia and Vida.

### Reagents

Monoclonal antibodies (mAbs) for flow cytometry: anti-FoxP3 (clone FJK-16S) conjugated to Phycoerythrin (PE)-Cyanine 7 (Cy7) and Allophycocyanin (APC), and anti-IFN-γ (clone XMG1.2) conjugated to PE-Cy7 were obtained from eBioscience (San Diego, CA, USA). Anti-CD4 (clone GK1.5) conjugated to APC and APC-Cy7; anti-CD25 (clone PC61) conjugated to Fluorescein isothiocyanate (FITC); anti-CD44 (clone IM7) conjugated to PE; anti-CD62L (clone MEL14) conjugated to APC-Cy7; anti-IL-17A (clone TC11-181710.1) conjugated to APC; anti-CD45.2 (clone 104) conjugated to PE-Cy7; anti-CD45.1 (clone A20) conjugated to Brilliant Violet (Bv)421; anti-CTLA4 (clone UC10-4139) conjugated to Bv421; anti-ICOS (clone C398.4A) conjugated to PE-Cy7; anti-CD39 (clone Duha59) conjugated to PE; anti-CD73 (clone Ty/11.8) conjugated to Peridin-Chlorophyll (PerCP); anti-CCR6 (clone 29-2L17) conjugated to APC; anti-glucocorticoid-induced tumor necrosis factor receptor-related protein (GITR; clone YGITR756) conjugated to PE-Cy7; anti-CD104 (clone 2E7) conjugated to Pacific Blue; anti-B220 (clone RA3-6B2) conjugated to PerCP; anti-TCRVα2 (clone B20.1) conjugated to PE and TCRVβ5 (clone MR9-4) conjugated to APC were purchased from Biolegend (San Diego, CA, USA). mAbs for Cell Culture: the followings mAbs low in endotoxins and azida free (LEAF) were purchased from Biolegend: anti-IL-4 (clone 11B11), anti-CD28 (clone 37.51), anti-CD3ε (clone 145-2C11), anti-IFN-γ (clone AN-18) and anti-IL-2 (clone JES6-1A12). Carrier-Free cytokines TGF-β3, TGF-β1, IL-6, IL-12, IL-23, IL-1β and IL-2 were purchased from Biolegend. Zombie Aqua™ Fixable Viability dye detectable by flow cytometry was purchased from Biolegend. Phorbol 12-myristate 13-acetate (PMA) and ionomycin were purchased from Sigma-Aldrich (St. Louis, MO, USA). Cell Trace Carboxyfluorescein succinimidyl ester (CFSE), Brefeldin A and Fetal Bovine Serum (FBS) were obtained from Life Technologies (Carlsbad, CA, USA). The peptide derived from myelin oligodendrocyte glycoprotein (pMOG_35–55_) was purchased from GeneTel Laboratories (Madison, WI, USA). The peptide derived from the chicken ovalbumin (OVA_323–339;_ OT-II peptide or pOT-II) was purchased from Genescript (Piscataway, NJ, USA). Freund’s Complete Adjuvant (CFA) was purchased from Thermo Scientific. Bovine Serum Albumin (BSA) was purchased to Rockland (Limerick, PA, USA).

### Generation of Bone Marrow Chimeras

Five-to-six weeks old B6.SJL-*Ptprc^a^* (*cd45.1*^+/+^) mice were lethally irradiated with two doses of 550 rad each (source of Co^60^) separated for 3 h. One-day later, irradiated mice received the i.v. transference of bone marrow precursors (10^7^ cells per mouse) obtained from WT (*cd45.2*^+/+^) or DRD5KO (*cd45.2*^+/+^) donor mice and maintained for 8 weeks with antibiotics (Trimethroprim and Sulfadoxine; Gorban 0.1% purchased from Merck Animal Health, Madison, NJ, USA) in the drinking water. The percentage of chimerism was determined as the percentage of CD45.2^+^ cells from the CD45^+^ leukocytes in peripheral blood, typically fluctuating between 80% and 85% (see Supplementary Figure S1). Bone marrow precursors were obtained from femurs and tibias as described before (Inaba et al., 1992).

### EAE Induction and Evaluation

Mice were injected s.c. with 50 μg pMOG (Genetel Laboratories, Madison, WI, USA) emulsified in complete Freund’s adjuvant (CFA; Invitrogen) supplemented with heat-inactivated Mycobacterium tuberculosis H37 RA (Difco Laboratories, Detroit, MI, USA). In addition, mice received 500 ng pertussis toxin (Calbiochem, La Jolla, CA, USA) i.p. on days 0 and 2. Clinical signs were assessed daily according to the following scoring criteria: 0, no detectable signs; 1, flaccid tail; 2, hind limb weakness or abnormal gait; 3, complete hind limb paralysis; 4, paralysis of fore and hind limbs; and 5, moribund or death. In some EAE experiments, total splenic CD4^+^ T-cells were purified by negative selection using magnetic beads-based kit (MACS^®^, Miltenyi Biotec, Bergisch Gladbach, Germany) and i.v. injected (5 × 10^6^ cells per mouse) 24 h before EAE induction. In other EAE experiments, splenic naive (CD25^−^CD62L^+^CD44^−^) CD4^+^ T-cells were purified by cell-sorting and i.v. injected (3 × 10^6^ cells per mouse) 24 h before EAE induction. In other experiments splenic Tregs (CD4^+^ CD25^high^ T-cells) were purified by cell-sorting and i.v. injected (7 × 10^5^ cells per mouse) 24 h before EAE induction.

### CD4^+^ T-Cell Isolation and Activation *in Vitro*

Total CD4^+^ T-cells were obtained by negative selection of splenocytes (MACS^®^). Naive (CD4^+^ CD25^−^) T-cell isolation was achieved by cell sorting using a FACS Aria II (BD, Franklin Lakes, NJ, USA), obtaining purities over 98%. Splenic DCs were purified using a CD11c+ magnetic selection kit (MACS^®^). All *in vitro* experiments were performed using complete IMDM medium (Life Technologies) 10% FBS. To assess proliferation, naive T-cells from OT-II mice were stained with CFSE (10 μM as indicated in figure legends) and cultured on a 5:1 (T-cells:DCs) ratio on U-bottom 96-well plates in the presence of OT-II peptide (OVA_323–339_, pOT-II; 200 ng/ml) for 2 or 3 days. T-cell activation was determined as IL-2 secretion in the co-culture supernatant by ELISA as previously described (González et al., 2013). The extent of T-cell proliferation was determined as the percentage of dilution of CFSE-associated fluorescence by flow cytometry.

### CD4^+^ T-Cell Differentiation *in Vitro*

Naive (CD4^+^CD62L^+^CD44^−^CD25^−^) T-cell were isolated by cell-sorting and stimulated with 50 ng/well of plate-bound anti-CD3 and 2 μg/mL soluble anti-CD28 Abs on flat-bottom 96-well plates (Thermo Scientific) for 5 days. To force the differentiation of the different Th phenotypes, naïve CD4^+^ T-cells were incubated in the conditions indicated above, in addition to a mixture of cytokines and blocking antibodies: Th1: 20 ng/mL IL-12, 10 ng/mL IL-2 and 5 μg/mL anti-IL-4; non-pathogenic Th17 (Th17np): during the first 2 days with 25 ng/mL IL-6, 5 ng/mL TGF-β1, 20 ng/mL IL-1β, 5 μg/mL anti-IL-4, 5 μg/mL anti-IL-2 and 5 μg/mL anti-IFN-γ and during the last 3 days with 25 ng/mL IL-6, 5 ng/mL TGF-β1; pathogenic Th17 (Th17p): during the first 2 days with 25 ng/mL IL-6, 5 ng/mL TGF-β3, 20 ng/mL IL-1β, 5 μg/mL anti-IL-4, 5 μg/mL anti-IL-2 and 5 μg/mL anti-IFN-γ and during the last 3 days with 25 ng/mL IL-6, 20 ng/mL IL-1β and 20 ng/mL IL-23. At different incubation times, cells were assessed for gene expression real time RT-PCR.

### Quantitative RT-PCR

Total RNA extracted from cells using the Total RNA EZNA kit (Omega Bio-Tek, Norcross, GA, USA), was DNase-digested using the TURBO DNA-free kit (Ambion, Thermo Fisher Scientific) and 1 μg of RNA was used to synthesize cDNA utilizing M-MLV reverse transcriptase, according to manufacturer’s instructions (Life Technologies). Quantitative gene expression analysis was performed using Brilliant II SYBR Green QPCR Master Mix (Agilent Technologies, Santa Clara, CA, USA), according to manufacturer’s recommendations. Primers were used at a concentration of 0.5 μM. We used 40 PCR cycles as follows: denaturation 10 s at 95°C, annealing 20 s at 60°C and extension 20 s at 72°C. Expression of target genes was normalized to *Gapdh*. The sequences of the primers used are indicated in Table 1.

**Table 1:** Primers sequences used for quantitative RT-PCR analyses.

Gene	Forward (5’ → 3’)	Reverse (5’ → 3’)	Reference
rorc	CAGAGGAAGTCAATGTGGGA	GTGGTTGTTGGCATTGTAGG	Fernández et al. (2016)
runx1	TGGCACTCTGGTCACCGTCAT	GAAGCTCTTGCCTCTACCGC	Logan et al. (2013)
runX3	CGACCGTTTGGAGACCTGC	GCGTAGGGAAGGAGCGGTCA	Wang et al. (2014)
tbx21	CCTGTTGTGGTCCAAGTTCAAC	CACAAACATCCTGTAATGGCTTGT	Smeltz et al. (1999)
il9	CTGATGATTGTACCACACCGTGC	GCCTTTGCATCTCTGTCTTCTGG	Li et al. (2014)
il10	GAAGACAATAACTGCACCCA	CAACCCAAGTAACCCTTAAAGTC	Fernández et al. (2016)
il17a	TTCATCTGTGTCTCTGATGCT	AACGGTTGAGGTAGTCTGAG	Fernández et al. (2016)
Il22	GACAGGTTCCAGCCCTA CAT	ATCGCCTTGATCTCTCCACT	Peng et al. (2014)
csf2	ACCACCTATGCGGATTTCAT	TCATTACGCAGGCACAAAAG	Kim et al. (2008)
grzb	ATCAAGGATCAGCAGCCTGA	TGATGTCATTGGAGAATGTCT	Fernández et al. (2016)
ifng	GAGCCAGATTATCTCTTTCTACC	GTTGTTGACCTCAAACTTGG	Fernández et al. (2016)
tgfb1	GCAACAACGCCATCTATGAG	TATTCCGTCTCCTTGGTTCAG	Fernández et al. (2016)
tgfb3	AGCGCACAGAGCAGAGAAT	GTCAGTGACATCGAAAGACAG	Yin et al. (2013)
drd5	CCCTAACATAACTCATCTTCTCC	TAACCCTGCAAGTTCATCCA	Franz et al. (2015)
gapdh	TCCGTGTTCCTACCCCCAATG	GAGTGGGAGTTGCTGTTGAAG	Prado et al. (2012)

### CD4^+^ T-Cell Proliferation and Differentiation *in Vitro*

To analyze T-cell proliferation *in vivo*, splenic CD4^+^ T-cells were isolated from OT-II mice, stained with 10 μM CFSE and then i.v. transferred (12.5 × 10^6^ cells/mouse) into WT recipient mice. One-day later mice were i.p. immunized with 25 μg OVA or just injected with vehicle (PBS). Three-days after immunization mice were sacrificed and spleen cells were isolated, immunostained with fluorochrome-conjugated antibodies for B220, CD4, TCR-Vα2 and TCR-Vβ5 and then CFSE-associated fluorescence was analyzed in the B220^−^ CD4^+^ TCR-Vα2^+^ TCR-Vβ5^+^ gate. The extent of T-cell proliferation was determined as the percentage of cells displaying dilution of CFSE-associated fluorescence or by the decrease of MFI in CFSE associated fluorescence in the CD4+ T-cell population expressing the transgenic OT-II TCR (TCR composed by Vα2 and Vβ5 chains) by flow cytometry. To determine the acquisition of inflammatory phenotypes by CD4+ T-cells *in vivo*, naive CD4^+^ CD25^−^ CD44^−^ CD62L^+^ T-cells were isolated from OT-II/*cd45.2*^+/+^ mice by cell-sorting and then i.v. transferred (10^5^ cells/mice) into WT/*cd45.1*^+/+^ recipient mice. One-day later, mice were s.c. immunized with 100 μg of pOT-II in CFA and the inflammatory phenotype of CD4^+^ T-cells in the draining lymph nodes (inguinal lymph nodes) was analyzed 7 and 14 days after immunization by intracellular cytokine staining of IL-17 and IFN-γ.

### Tregs Suppression Assay

Splenic naive CD4^+^ CD25^−^ T-cells were isolated from OT-II mice by cell-sorting, stained with 5 μM CFSE and then co-cultured with splenic DCs on a 5:1 (T-cells:DCs) ratio on U-bottom 96-well plates in the presence of OVA (5 μM) for 5 days. Splenic CD4^+^ CD25^high^ T-cells (nTregs) were isolated from OT-II mice by cell-sorting and added to the co-cultures at indicated ratios. The extent of proliferation of naive T-cells (effector T cells; Teffs) was determined as the percentage of cells displaying dilution of the CFSE-associated fluorescence. One-hundred percent proliferation corresponded to the extent of proliferation of Teffs in the absence of nTregs (positive control). % Suppression was calculated as 100% proliferation (from positive control) minus % proliferation observed for each sample.

### Flow Cytometry

For analysis of cytokine production, cells were restimulated with 1 μg/mL ionomycin and 50 ng/mL PMA in the presence of 5 μg/mL brefeldin A for 4 h. Cell surface staining was carried out in PBS with 2% FBS. For intracellular staining, cells were first stained with Zombie Aqua Fixable Viability kit (Biolegend), followed by staining for cell-surface markers and then resuspended in fixation/permeabilization solution (3% BSA and 0.5% saponin in PBS). Samples including the analysis of Foxp3 transcription factor were resuspended in fixation/permeabilization solution (Foxp3 Fixation/Permeabilization; eBioscience) according to the manufacturer instructions. To determine absolute number of cells, samples were analyzed in the presence of 123count eBeads (eBioscience) according to manufacturer’s guidelines. Data were collected with a Canto II (BD) and results were analyzed with FACSDiva (BD) and FlowJo software (Tree Star, Ashlan, OR, USA).

### Statistical Analysis

Differences in means between two groups (different genotype or different conditions) were analyzed by unpaired 2-tailed Student’s *t*-test. Progression of EAE severity curves were compared with a non-parametric Mann–Whitney rank sums two-tailed *U* test. *P* value ≤ 0.05 was considered significant. Analyses were performed with GraphPad Prism 6 software.

### Ethics Statement

This study was carried out in accordance with the recommendations of the institutional guidelines of Fundación Ciencia & Vida. The protocol was approved by the Bioethics and Biosecurity committee of the Fundación Ciencia & Vida.

## Results

### DRD5-Signaling in Naive CD4^+^ T-Cells Favors the Development of the Inflammatory Response Associated to EAE

**Figure 1:**
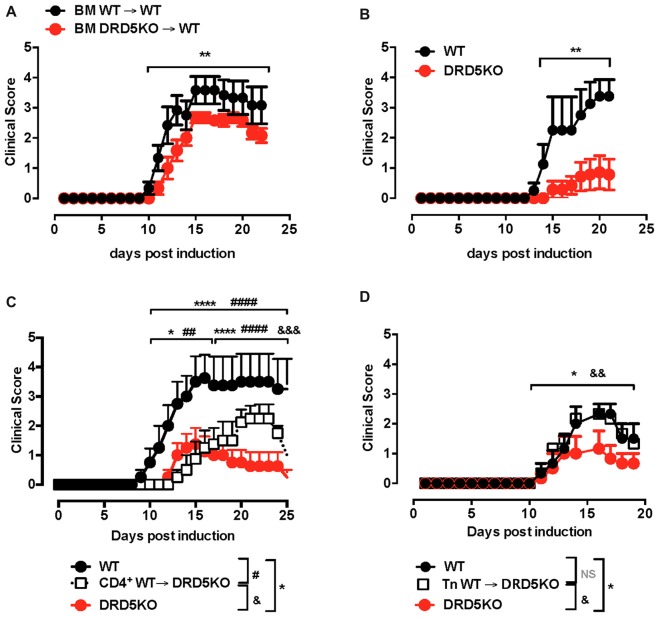
DRD5-signaling in naive CD4^+^ T-cells favors the development of the inflammatory response associated to experimental autoimmune encephalomyelitis (EAE). **(A)** Wild-type (WT) *cd45.1*^+/+^ mice were lethally irradiated and then bone marrow (10^7^ cells/per mouse) from WT *cd45.2*^+/+^ (black symbols) or DRD5KO *cd45.2* ^+/+^ (red symbols) mice were i.v. transferred to reconstitute the hematopoietic compartment. Eight-week later, EAE was induced by immunization with pMOG in freund’s complete adjuvant (CFA) followed by *Pertussis* Toxin injection and disease severity was daily determined throughout the time-course of disease development. Values represent mean ± SEM with *n* = 5–6 mice per group. **p < 0.01 by Mann-Whitney *U*-test. **(B)** EAE was induced in WT (black symbols) and DRD5KO (red symbols) mice by immunization with pMOG in CFA followed by *Pertussis* Toxin injection and disease severity was daily determined throughout the time-course of disease development. Values represent mean ± SEM with *n* = 4–7 mice per group. **p < 0.01 by Mann-Whitney *U*-test. **(C)** Total splenic CD4^+^ T-cells were isolated from WT mice by negative selection using magnetic beads-based kit (from Miltenyi) and then they were i.v. injected into DRD5KO mice (5 × 10^6^ cells/per mouse; white symbols). One-day later, EAE was induced by immunization with pMOG in CFA followed by *Pertussis* Toxin injection and disease severity was daily determined throughout the time-course of disease development. EAE was also induced in WT (black symbols) and untransferred DRD5KO (red symbols) mice as control groups. Values represent mean ± SEM with *n* = 4 mice per group. *p < 0.05 and ****p < 0.0001 WT vs. untransferred DRD5KO mice; ^##^*p* < 0.01 and ^####^*p* < 0.0001 WT vs. transferred DRD5KO; ^&&&^*p* < 0.001 transferred vs. untransferred DRD5KO mice by Mann-Whitney *U*-test. **(D)** Naive CD4^+^ T-cells (CD4^+^ CD25^−^ CD62L^+^ CD44^−^) were isolated from WT mice by cell-sorting and then they were i.v. injected into DRD5KO mice (3 × 10^6^ cells/per mouse; white symbols). One-day later, EAE was induced by immunization with pMOG in CFA followed by *Pertussis* Toxin injection and disease severity was daily determined throughout the time-course of disease development. EAE was also induced in WT (black symbols) and untransferred DRD5KO (red symbols) mice as control groups. Values represent mean ± SEM with *n* = 3–4 mice per group. *p < 0.05 WT vs. untransferred DRD5KO mice; ^&&^*p* < 0.01 transferred vs. untransferred DRD5KO mice by Mann-Whitney *U*-test.

Since we previously appreciated a difference in the severity of EAE manifestation between animals deficient in DRD5 confined to DCs and animals displaying a global deficiency of DRD5 (Prado et al., 2012), we wondered whether DRD5-signaling in other immune cells was relevant in the regulation of the inflammatory response involved in EAE. To address this question, we first compared how was the development of EAE in animals displaying a global deficiency of DRD5 with animals bearing DRD5-deficiency confined to the hematopoietic compartment. For this purpose, we generated chimeric animals by killing bone marrow of WT recipient animals with a myeloablative dose of γ-irradiation and then transferring WT or DRD5-deficient bone marrow. Eight weeks after bone marrow transplantation, we obtained chimeric animals bearing WT or DRD5-deficient blood cells and WT genetic background outside the hematopoietic compartment (Supplementary Figure S1). When EAE was induced in these chimeric animals we observed that DRD5-deficiency confined to bone marrow resulted in attenuated EAE manifestation (Figure 1A), although the reduction in EAE severity was less marked than attenuation observed between WT and DRD5KO mice (Figure 1B). Of note, this lower difference in EAE manifestation observed between chimeric animals bearing WT or DRD5-deficient bone marrow may be due to the fact that the percentage of chimerism was around 80%–85% but not complete (Supplementary Figure S1). For this reason and also considering that autoimmune response in EAE is driven by CD4^+^ T-cells, which have been described to express DRD5 (Franz et al., 2015), we next carried out gaining of function experiments by performing adoptive transfer of CD4^+^ T-cells. In this regard, we hypothesized that if DRD5-signaling in CD4^+^ T-cells is relevant in contributing to the inflammation associated to EAE, we should rescue the phenotype of DRD5KO mice in EAE upon transfer of WT CD4^+^ T-cells. Accordingly, our results show that when DRD5KO mice received the transference of WT CD4^+^ T-cells, EAE severity was significantly stronger than DRD5KO mice, although still in a lesser extent than EAE severity observed in WT mice (Figure 1C). Since Tregs, a subpopulation of CD4^+^ T-cells, play a relevant role attenuating EAE development in late stages of the disease manifestation (Petermann et al., 2010) and they also express DRD5 (Kipnis et al., 2004; Cosentino et al., 2007), we next wanted to evaluate the effect of DRD5-signaling confined to CD4^+^ T-cells without the potential contribution of DRD5-signaling from Tregs. For this purpose, we next performed a set of experiments in which we isolate naive CD25^−^ CD4^+^ CD44^−^ CD62L^+^ T-cells from WT mice, a subset devoid of Tregs cells (CD25^high^ CD4^+^), and then these cells were transferred into DRD5KO recipient mice just before EAE induction. The results show that the transfer of DRD5-sufficient naive CD4^+^ T-cells, in the absence of Tregs, into DRD5-defficient animals results in a complete rescue in EAE manifestation, displaying similar EAE severity than observed for WT mice (Figure 1D). Thereby, these results indicate that DRD5-signaling in naive CD4^+^ T-cells exerts a pro-inflammatory role favoring EAE development.

### DRD5-Signaling in CD4^+^ T-Cells Potentiates T-Cell Activation

**Figure 2:**
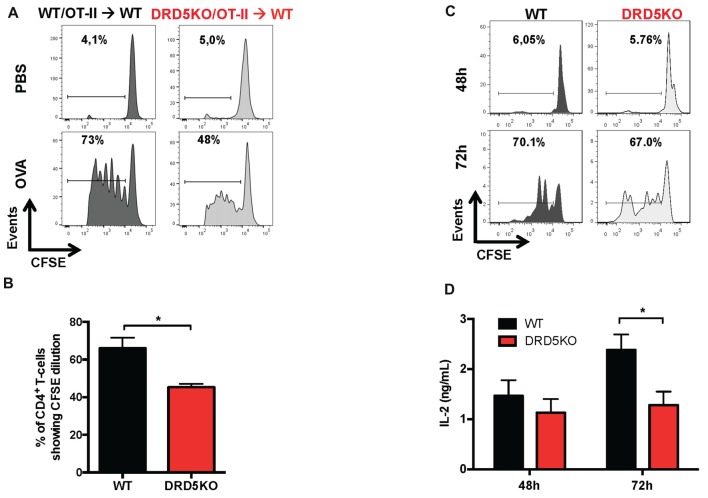
DRD5-signaling in CD4^+^ T-cells potentiates T-cell activation. **(A,B)** CD4^+^ T-cells were isolated from WT/OT-II or DRD5KO/OT-II mice by negative selection using magnetic beads-based kit (from Miltenyi), stained with 10 μM carboxyfluorescein succinimidyl ester (CFSE) and then i.v. transferred (12.5 × 10^6^ cells/mice) into WT recipient mice. One-day later mice were i.p. immunized with 25 μg ovalbumin (OVA) or just injected with vehicle (PBS). Three-day after immunization mice were sacrificed and spleen cells were isolated, immunostained with fluorochrome-conjugated antibodies for B220, CD4, TCR-Va2 and TCR-Vβ5 and then CFSE-associated fluorescence was analyzed in the B220^−^ CD4^+^ TCR-Va2^+^ TCR-Vβ5^+^ gate. **(A)** Representative histograms of CFSE-associated fluorescence vs. number of cells (events) are shown. Markers delimiting proliferating cells are shown. Percentages of CD4^+^ T-cells in the region of proliferating cells are indicated. **(B)** Percentages of CD4^+^ T-cells in the proliferating area from three independent experiments are represented in the bars graphs. Values represent mean ± SEM. *p < 0.05 by unpaired Student’s *t*-test. **(C,D)** Naive CD4^+^ CD25^−^ CD62L^+^ CD44^−^ T-cells were isolated from WT/OT-II or DRD5KO/OT-II mice by cell-sorting, stained with 10 μM CFSE and then co-cultured with splenic Dendritic cells (DCs) isolated by positive CD11c^+^ selection using magnetic beads based kit (from Miltenyi) at a ratio of CD4+ T-cells:DCs = 5:1, in the presence of pOT-II (200 ng/ml) for 48 h or 72 h. **(C)** Cells were immunostained with fluorochrome-conjugated antibodies for CD4, TCR-Va2 and TCR-Vβ5 and then CFSE-associated fluorescence was analyzed in the CD4^+^ TCRVa2^+^ TCRVβ5^+^ gate. Representative histograms of CFSE-associated fluorescence vs. number of cells (events) are shown. Markers delimiting proliferating cells are shown. Percentages of CD4^+^ T-cells in the region of proliferating cells are indicated. **(D)** IL-2 production was determined in the culture supernatant by ELISA. Data from three independent experiments is shown. Values represent mean ± SEM. *p < 0.05 by unpaired Student’s *t*-test.

To address the question of how DRD5-signaling favors the inflammatory potential of CD4^+^ T-cells we next evaluated the role of this receptor in T-cells activation. Accordingly, we first determined the role of DRD5-signaling in the proliferation of CD4^+^ T-cells *in vivo*, which is directly related with the potency of T-cell activation. For this purpose, we performed adoptive transfer experiments using CD4^+^ T-cells from OT-II transgenic mice, which express a transgenic T-cell receptor (TCR) specific for the recognition of a peptide derived from the chicken ovalbumin (OVA_323–339_; pOT-II). To determine T-cell activation *in vivo*, we isolate CD4^+^ T-cells from WT/OT-II or from DRD5KO/OT-II mice, which were loaded with the fluorescent probe CFSE and then transferred into WT recipient mice. Subsequently, transferred mice were immunized with pOT-II in CFA and the potency of proliferation induced in pOT-II-specific T-cells was determined 3 days later in the spleen as the extent of the dilution of CFSE-associated fluorescence as previously described (Contreras et al., 2016). The results show that DRD5-deficiency resulted in attenuated CD4^+^ T-cell proliferation as indicated by a lower percentage of OT-II cells displaying dilution of CFSE-associated fluorescence (Figures 2A,B). To obtain further evidence in the role of DRD5-signaling in CD4^+^ T-cell activation, we next performed *in vitro* experiments in which antigen-specific T-cell activation was induced by incubating naive CD4^+^ T-cells isolated from WT/OT-II or DRD5KO/OT-II in the presence or WT DCs loaded with OVA. To evaluate the potency of T-cell activation, we determined the extent of proliferation as the level of CFSE dilution and the secretion of IL-2 into the culture supernatant, which corresponds to the main T-cell growth factor. The results show that DRD5-deficiency in CD4^+^ T-cells not only resulted in decreased proliferation (Figure 2C) but also in reduced IL-2 production (Figure 2D). Thus, these results indicate that DRD5-signaling potentiates CD4^+^ T-cell activation *in vitro* and *in vivo*.

### DRD5-Signaling in CD4^+^ T-Cells Favors the Acquisition of Th17 Phenotype

**Figure 3:**
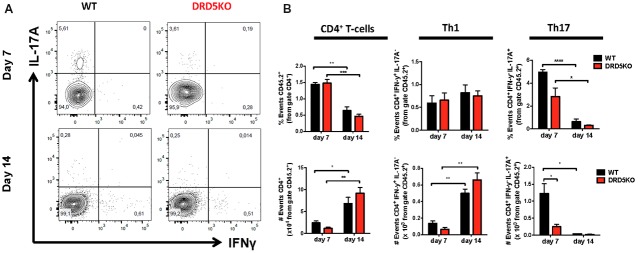
DRD5-signaling in CD4^+^ T-cells favors the acquisition of Th17 phenotype. **(A,B)** Naive CD4^+^ CD25^−^ T-cells were isolated from WT/OT-II/*cd45.2*^+/+^ or DRD5KO/OT-II/*cd45.2*^+/+^ mice by cell-sorting and then i.v. transferred (10^5^ cells/mouse) into WT/*cd45.1*^+/+^ recipient mice. One-day later, mice were s.c. immunized with 100 μg of pOT-II in CFA and CD4^+^ T-cells were analyzed by flow cytometry in the draining lymph nodes (inguinal lymph nodes) 7 and 14 days after immunization. **(A)** Representative contour-plots analyzing IFN-γ vs. IL-17 in the CD45.2^+^ CD4^+^ gated population. Numbers in each quadrant represent the percentage of cells in the corresponding quadrant. **(B)** Quantification of the percentage (top panels) and absolute number (bottom panels) of total CD4^+^ T-cells (left panels), Th1 cells (central panels) and Th17 cells (right panels) contained in the CD45.2^+^ population. Values represent mean ± SEM with *n* = 4 mice per group. *p < 0.05; **p < 0.01; ***p < 0.001; ****p < 0.0001 by unpaired Student’s *t*-test.

**Figure 4:**
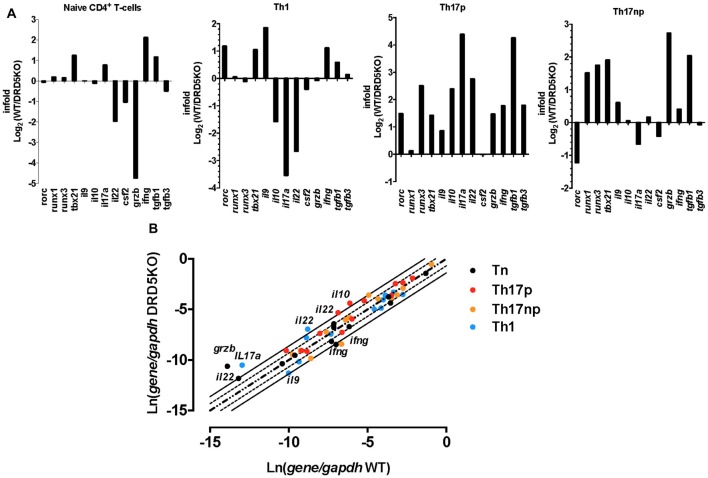
DRD5-deficiency alters the transcriptional pattern of different effector phenotypes of CD4^+^ T-cells. Splenic naive (CD4^+^ CD62L^+^ CD44^−^CD25^−^) T-cell were isolated from WT or DRD5KO mice by cell-sorting and immediately analyzed (Tn) or stimulated with anti-CD3 and anti-CD28 Abs in the presence of biased conditions to differentiate to Th1, Th17p or Th17np for 5 days and then analyzed. Afterward, total RNA was extracted and the transcription of a panel of relevant genes was analyzed by qRT-PCR. *Gapdh* transcription was used as house keeping. **(A)** Data represented as infold (WT relative to DRD5KO) indicating how many times the levels of mRNA obtained in WT duplicates those obtained in DRD5KO. **(B)** Data represented as Ln of mRNA levels/house keeping (WT vs. DRD5KO). Central diagonal dotted line indicates same mRNA levels. External dotted lines indicate 2-fold mRNA levels and external solid lines indicate 4-fold mRNA levels. **(A,B)** Values are the mean from three independent experiments.

To gain deeper insight in the role of DRD5-signaling in the CD4^+^ T-cell response, we next determined how this receptor affects the differentiation of naive CD4^+^ T-cells into effector phenotypes. Because the Th1 and Th17 are the main subsets of effector T-cells (Teffs) driving the inflammatory response involved in EAE (Prado et al., 2013) and both of them express DRD5 transcripts (Supplementary Figure S2), we next evaluated the potential role of DRD5-signaling in the acquisition of these functional phenotypes. For this purpose, we performed experiments in which naive CD4^+^ T-cells were isolated from WT/OT-II or from DRD5KO/OT-II mice and transferred into WT recipients. Afterward, these recipient mice were subcutaneously immunized with pOT-II and then the extent of acquisition of Th1 and Th17 functional phenotypes by the transferred CD4^+^ T-cells was analyzed in the draining lymph nodes at different time points. The results show that despite there were no significant differences in the absolute number and frequency of Th1 cells (see IFN-γ^+^ IL-17^−^ T-cells in Figure 3A and in Figure 3B middle panels), DRD5-deficiency in CD4^+^ T-cells resulted in a strong reduction in the absolute number of Th17 cells in the draining lymph nodes 1 week after immunization (see IFN-γ^−^ IL-17^+^ T-cells in Figures 3A and in Figure 3B right-bottom panel). DRD5-deficiency in CD4^+^ T-cells resulted also in a trend of decreased Th17 frequency in the draining lymph nodes 1 week after immunization, although this difference was not statistically significant (Figure 3B right-top panel). To evaluate whether difference in the acquisition of functional phenotypes observed between DRD5-deficient and DRD5-suficient CD4^+^ T-cells was due to altered viability of these cells, we determined the percentage of living cells among transferred CD4^+^ T-cells. The analyses show that frequencies of viable cells were similar in both genotypes, ruling out this possibility (Supplementary Figure S3A). Interestingly, irrespective of the genotype of CD4^+^ T-cells, the absolute number of Th1 OT-II CD4^+^ T-cells in the draining lymph nodes was increased after 2 weeks (Figure 3B middle-bottom panel), whilst the number of Th17 OT-II CD4^+^ T-cells was strongly reduced (Figure 3B right-bottom panel), which is probably explained by the faster exit of Th17 cells from the draining lymph nodes after activation (Dardalhon et al., 2008; Reboldi et al., 2009). It is also noteworthy that, irrespective of their phenotype and genotype, the frequency of total OT-II CD4^+^ T-cells transferred is reduced in the draining lymph nodes 2 weeks after immunization (Figure 3B left-top panel) even when the absolute number is increased (Figure 3B left-bottom panel). This fact is likely to be due to the activation of endogenous CD4^+^ T-cells (CD45.1^+^) with specificity for pOT-II or for different antigens from mycobacterium tuberculosis contained in the CFA used as adjuvant in these experiments. To further explore the role of DRD5-signaling in Teffs, we quantified the transcriptional levels of a panel of different cytokines and transcription factors involved in the effector function of Th1 and Th17 cells. For this purpose, we performed a quantitative RT-PCR array of a set of relevant functional molecules in naive CD4^+^ T-cells, Th1 and Th17 cells obtained from WT or DRD5KO mice. To gain a deeper insight in the role of DRD5-signaling in Th17 function, we evaluated the transcriptional panel of these molecules in Th17 cells differentiated in pathogenic (Th17p) or non-pathogenic (Th17np) conditions (Lee et al., 2012). The results show that DRD5-deficiency results in decreased IFN-γ transcription by naive CD4^+^ T-cells or Th17np (Figure 4A left and right panels, and Figure 4B), increased transcription of IL-10 and IL-22 by Th17p (Figure 4A middle-right panel, and Figure 4B), exacerbated transcription of IL-17 and IL-22 by Th1 (Figure 4A middle-left panel, and Figure 4B) and increased transcription of granzyme B and IL-22 by naive CD4^+^ T-cells (Figure 4A left panel, and Figure 4B). Of note, we observed similar frequency of viable cells after CD4^+^ T-cell differentiation (Supplementary Figure S3B), ruling out the possibility that differential expression of cytokines and transcription factors was due to altered cell viability. Taken together, these results suggest that DRD5-defficiency in CD4^+^ T-cells results in impaired acquisition of the inflammatory Teff phenotypes and reduced expansion of Th17 cells.

### DRD5-Signaling in Tregs Favors Their Suppressive Activity

**Figure 5:**
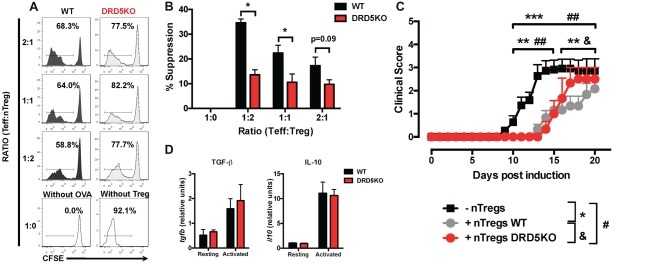
DRD5-signaling in Tregs favors their suppressive activity *in vitro* and *in vivo*. **(A,B)** Splenic CD4^+^ CD25^−^ T-cells (Teff) were isolated from WT/OT-II mice by cell-sorting, stained with 5 μM CFSE and then co-cultured with splenic DCs isolated from WT mice by positive CD11c^+^ selection using magnetic beads based kit (MACS) at a ratio of CD4+ T-cells:DCs = 5:1, in the presence of OVA (5 μM) for 5 days. Splenic CD4^+^ CD25^high^ T-cells (nTregs) were isolated from WT/OT-II or DRD5KO/OT-II mice by cell-sorting and added to the co-cultures at indicated ratios. **(A)** Representative histograms of CFSE-associated fluorescence vs. number of cells (events) in the CD4^+^ TCR-Va2^+^ TCR-Vβ5^+^ gate are shown. Markers delimiting proliferating cells are indicated. Percentage of CD4^+^ T-cells in the region of proliferating cells are indicated. **(B)** Percentages of suppression of Teff proliferation from three independent experiments are represented in the bars graphs. One-hundred percent proliferation corresponds to the extent of proliferation of Teffs in the absence of nTregs (positive control). % Suppression is calculated as 100% proliferation (from positive control) minus % proliferation observed for each sample. Values represent mean ± SEM. *p < 0.05 by unpaired Student’s *t*-test. **(C)** Splenic CD4^+^ CD25^high^ T-cells (nTregs) were isolated from WT (gray symbols) or DRD5KO (red symbols) mice by cell-sorting and i.v. transferred into WT recipient mice (7 × 10^5^ cells per mouse). One-day later, EAE was induced by immunization with pMOG in CFA followed by *Pertussis* Toxin injection and disease severity was daily determined throughout the time-course of disease development. EAE was also induced in untransferred WT (black symbols) as a control group. Values represent mean ± SEM with *n* = 3–12 mice per group. ^##^*p* < 0.01 untransferred WT vs. DRD5KO nTregs transferred into WT mice; **p < 0.01 and ***p < 0.001 untransferred WT vs. WT nTregs transferred into WT mice; ^&^*p* < 0.05 WT nTregs transferred into WT mice vs. DRD5KO nTregs transferred into WT mice by Mann-Whitney *U*-test. **(D)** Splenic CD4^+^ CD25^high^ T-cells (nTregs) were isolated from WT or DRD5KO mice by cell-sorting and immediately analyzed (resting) or activated with anti-CD3 and anti-CD28 antibodies in the presence of IL-2 for 36 h (activated). Afterward, total RNA was extracted and the levels of transcripts for TGF-β and IL-10 were quantified by real time RT-PCR. Data is represented as the levels of transcripts relative to the house-keeping *gapdh*. Values represent mean ± SD from at least three independent experiments. No significant differences were found between different genotypes.

**Figure 6:**
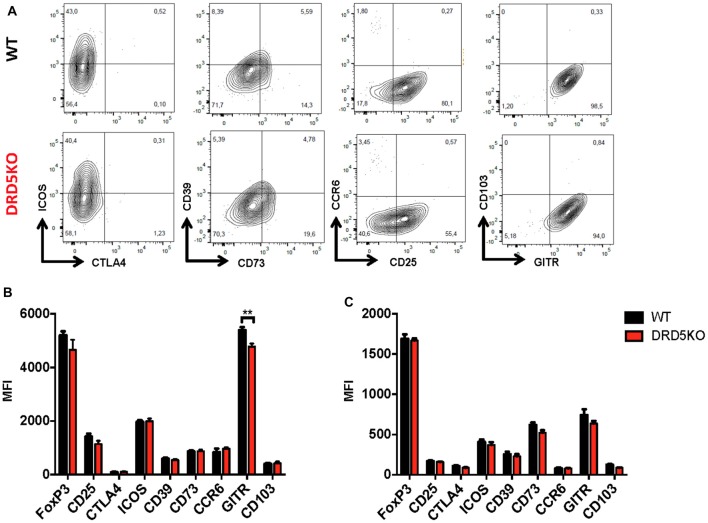
DRD5-signaling favors glucocorticoid-induced tumor necrosis factor receptor-related protein (GITR) expression in Tregs. Splenic CD4^+^ CD25^high^ T-cells (nTregs) were isolated from WT or DRD5KO mice by cell-sorting and immediately analyzed (resting) or activated with anti-CD3 and anti-CD28 antibodies in the presence of IL-2 for 36 h (activated). Cells were immunostained for a panel of surface markers associated to Tregs function, then permeabilized and immunostained for Foxp3 and analyzed by flow cytometry. **(A)** Representative dot plots are shown. Percentage of cells in each quadrant associated to immunostaining of each molecule is indicated. Data from activated **(B)** and resting **(C)** Tregs are represented as MFI. Values represent mean ± SD from at least four independent experiments. **p < 0.01 by unpaired Student’s *t*-test.

Since we observed that the transference of DRD5-sufficient total CD4^+^ T-cells into DRD5KO mice just partially rescues the phenotype on EAE manifestation (Figure 1C), whilst the transference of DRD5-sufficient naive CD25^−^ CD4^+^ CD44^−^ CD62L^+^ T-cells into DRD5KO mice completely rescues the phenotype on EAE manifestation (Figure 1D), we next wondered whether DRD5-signaling plays a relevant role in the Tregs (CD25^high^ CD4^+^) response. To address this question, we first determined the effect of DRD5-signaling in the suppressive activity of Tregs. For this purpose, we performed *in vitro* assays in which WT naive OT-II CD4^+^ T-cells (Teff) were loaded with CFSE, activated with OVA-loaded DCs and co-incubated with increasing concentrations of DRD5-defficient or DRD5-sufficient OT-II Tregs and the extent of inhibition of Teff proliferation was quantified. The results show that DRD5-sufficient Tregs exert a stronger inhibition of Teff proliferation than that exerted by DRD5-defficient Tregs (Figures 5A,B), thus indicating that DRD5-signaling favors a stronger suppressive activity by Tregs. To evaluate whether DRD5-signaling in Tregs plays a relevant role in the suppressive activity *in vivo*, we next performed experiments in which DRD5-sufficient or DRD5-defficient Tregs were transferred into WT recipient mice just before EAE induction. In the same direction of conclusions obtained from *in vitro* experiments (Figures 5A,B), these results show that EAE severity was reduced by DRD5-sufficient Tregs in a higher extent than that exerted by DRD5-defficient Tregs (Figure 5C), thus indicating that DRD5-signaling in Tregs favors a more potent suppressive activity *in vivo*. It is noteworthy that there was not differences in the extent of attenuation of EAE manifestation exerted by DRD5-sufficient and DRD5-defficient Tregs until late stages (after day 15 post-induction) in the time-course of EAE development, indicating that DRD5-signaling in Tregs was relevant only in late stages of EAE manifestation. To gain insight in the mechanism of how DRD5-signaling favors the suppressive activity of Tregs, we next evaluated the transcriptional level of the main cytokines associated to Tregs function, IL-10 and TGF-β, however we did not find any difference between DRD5-sufficient and DRD5-defficient Tregs (Figure 5D). To confirm whether IL-10 production was not affected by DRD5-signaling in Tregs we also analyzed the extent of IL-10 production at the level of protein and we found no differences between WT and knockout cells (Supplementary Figure S4). To further explore how DRD5-signaling potentiates the suppressive activity of Tregs, we next evaluated the level of protein expression of a panel of surface molecules and transcription factor associated with the suppressive activity of Tregs. The results show that the only difference detected between DRD5-sufficient and DRD5-defficient Tregs was that DRD5-deficiency results in a significant reduction in the expression of GITR (Figures 6A,B), a molecule that has been related with the expansion of Tregs (Ronchetti et al., 2015). Of note, this difference in GITR expression was observed only in activated Tregs (Figure 6B), but not in resting conditions (Figure 6C). Thus, these results together indicate that DRD5-signaling in Tregs plays a relevant role favoring Tregs-mediated suppressive response, an effect that would be exerted at least in part by increasing the expression of GITR.

## Discussion

Our findings here described represent the first genetic evidence indicating a relevant role of DRD5-signaling in the CD4^+^ T-cell response involved in EAE. Interestingly these findings suggest a dual role of DRD5-signaling, which are manifested at different stages during the time-course of EAE development and trigger opposite outcomes. Whereas the stimulation of DRD5 in naive CD4^+^ T-cells potentiates the inflammatory Th17-driven response early after the onset of the disease, DRD5-signaling in Tregs favors the suppressive activity of these cells dampening inflammation just once the peak of disease manifestation has been reached. Thus, these findings contribute to a better understanding of the complex regulation of the CD4^+^ T-cell driven autoimmune response involved in EAE and also illustrate how depending on the timing and on the precise T-cell subset, neuroimmune communications may exert different outcomes.

In a previous study, we showed genetic and pharmacologic evidence indicating that DRD5-signaling in CD4^+^ T-cells favored T-cell activation *in vitro*, a process that was mediated by ERK1/2 phosphorylation (Franz et al., 2015). In the same study we appreciated that DRD5-signaling in CD4^+^ T-cells had no effect in the process of Th1 differentiation *in vitro*. According with this previous study, our present results indicate that DRD5-signaling is relevant *in vivo* favoring T-cell activation (Figure 2) and without effect in the acquisition of the Th1 phenotype (Figure 3). Moreover, in agreement with our results about the role of DRD5-signaling in the effector CD4^+^ T-cell response *in vivo*, previous studies have shown pharmacologic evidence suggesting that the systemic antagonism of type I DRs attenuates the potency of Th17-driven responses (Nakano et al., 2008, 2011; Hashimoto et al., 2009; Okada et al., 2009; Nakagome et al., 2011; Nakashioya et al., 2011). In this regard, the systemic administration of the type I DRs antagonist SCH23390 reduces the clinical manifestation of EAE in mice immunized with a peptide derived from myelin proteolipid protein (Nakano et al., 2008). Similarly, subsequent studies showed that the same drug attenuates the inflammatory response in collagen-induced arthritis (Nakano et al., 2011; Nakashioya et al., 2011), allergic asthma (Nakagome et al., 2011; Gong et al., 2013), diabetes (Hashimoto et al., 2009) and nephrotoxic serum nephritis (Okada et al., 2009). Mechanistic analyses suggested that the therapeutic effect exerted by SCH23390 in these inflammatory disorders is due to the type I DRs antagonism in both CD4^+^ T-cells and DCs. Addressing the molecular mechanism involved in type I DRs signaling in CD4^+^ T-cells, the evidence suggests that type I DRs stimulation promotes B-cell activating transcription factor activity, favoring RORγt up-regulation and consequently Th17-mediated responses, which is inhibited by SCH23390 (Gong et al., 2013). Nevertheless, the pharmacologic approach used in all those studies does not allow the discrimination between the effects mediated by DRD5 or DRD1 since the drug inhibits both type I DRs with similar affinities (Bourne, 2001). Here we present genetic evidence indicating an important role of DRD5-signaling in promoting the acquisition of Th17 inflammatory phenotype *in vivo* (Figure 3).

Two previous studies have addressed the role of dopaminergic regulation of Tregs function (Kipnis et al., 2004; Cosentino et al., 2007). In apparent controversy with the present work, the studies carried out by Kipnis et al. (2004) and Cosentino et al. (2007) indicated that type I DRs stimulation attenuates Tregs suppressive activity by using a pharmacologic approach. Nonetheless, these studies were performed with drugs that cannot discriminate between their effects mediated by DRD1 or DRD5, as those drugs display very similar affinities for both receptors (Kipnis et al., 2004; Cosentino et al., 2007). Thus, it is likely that the inhibitory effect of dopamine or dopaminergic analogs on Tregs activity appreciated in those studies was mediated by DRD1 rather than DRD5. Here, we show genetic evidence indicating unequivocally that DRD5-deficiency in Tregs results in impaired suppressive activity of these cells *in vitro* and *in vivo* (Figure 5).

With these results in mind arise the question of where and when dopamine is relevant as a source available for CD4^+^ T-cells. In this regard, relevant sources of dopamine have been described in secondary lymphoid organs, the organs where T-cell responses are initiated, as well as in some target organs, where effector CD4^+^ T-cells promote inflammation. For instance, secondary lymphoid organs such as lymph nodes and the spleen are highly innervated by sympathetic fibers immunoreactive for dopamine (Weihe et al., 1991; Haskó and Szabó, 1998). In this way, the peripheral nervous system might exert regulation of the initiation of T-cell responses (Ben-Shaanan et al., 2016). According to this possibility, the present results (Figures 2, 3) and previous studies (Pacheco et al., 2014) suggest that dopamine produced in draining lymph nodes shapes the expansion and differentiation of naive CD4^+^ T-cells toward the different functional T-cell phenotypes. In addition to the sympathetic fibers, DCs and follicular helper T-cells (T_FH_) may also represent important sources of dopamine in secondary lymphoid organs. Indeed, we have recently described how dopamine contained in DCs plays an important role potentiating Th1 and Th17 responses involved in EAE (Prado et al., 2012, 2018). Furthermore, Papa et al. (2017) recently described how T_FH_ produce high amounts of dopamine and release it upon cognate interaction with B-cells in the germinal center, thus accelerating the production of high-affinity antibodies by plasma-cells. Thereby, dopamine produced in different specific locations inside secondary lymphoid organs might strengthen the initiation and development of adaptive immunity. With regard to the organs target of inflammation as potential relevant sources of dopamine for regulation of effector CD4^+^ T-cell response, altered levels of dopamine in different structures of the brain have been associated with neuroinflammation involved in some neurodegenerative disorders. For instance, dopamine levels have been found to be dramatically reduced in the nigrostriatal pathway of Parkinson’s disease patients as well as in animal models of this disorder (Ehringer and Hornykiewicz, 1960; Brochard et al., 2009). We have previously suggested that this pathologic reduction in dopamine levels results in the selective stimulation of the DR displaying the highest affinity for dopamine, the DRD3 (Pacheco, 2017). In this regard, we have previously found that DRD3-signaling triggers a strong inflammatory behavior of CD4^+^ T-cells that infiltrate the brain in an animal model of Parkinson’s diseases favoring the neurodegenerative process in the nigrostriatal pathway (González et al., 2013). On the other hand, EAE, the animal model used here to study MS and the autoimmune response associated, involves an increase of dopamine levels in the striatum during the peak of the disease (Balkowiec-Iskra et al., 2007). Since we observed a role of DRD5 in Tregs only once the peak of EAE manifestation has been reached, our results here suggest that the increase of striatal dopamine would be relevant stimulating DRD5 in Tregs, thus favoring their suppressive activity and dampening disease manifestation (Figure 5). Another potential source of dopamine present in the tissues target of inflammation may be Tregs. According to this idea, Tregs represent an important CD4^+^ T-cell subset infiltrating the brain and spinal cord during late stages of EAE manifestation, which is associated with the recovery phase of the disease (Petermann et al., 2010). Moreover, these cells have been described to synthesize and store important amounts of dopamine, which can be released upon treatment with reserpine (Cosentino et al., 2007). Despite the endogenous stimulus triggering dopamine release from Tregs has not been found yet, IFN-β has been proposed as a plausible candidate (Cosentino et al., 2012). Taken together these studies suggest that dopamine produced in the secondary lymphoid organs may strengthen the initial development of adaptive immune responses, whilst dopamine present in the inflamed target tissue may shape the effector and suppressive T-cell response depending in the timing and in the precise levels of available dopamine.

Interestingly, not only the CNS may represent a target tissue of inflammation with relevant sources of dopamine, but also the gut mucosa. In this regard, the gut mucosa has been found to be a major source of dopamine in healthy conditions, a source that is strongly reduced upon inflammation (Magro et al., 2002, 2004; Asano et al., 2012). Previous evidence has suggested that in healthy conditions high dopamine levels in the gut promote the stimulation of the low affinity DRs, specially the DRD2, whilst under inflammatory conditions low dopamine levels favors the selective stimulation of DRD3 (Pacheco, 2017). Furthermore, whereas DRD3-stimulation has been shown to promote pro-inflammatory CD4^+^ T-cell responses in the gut mucosa, including Th1 and Th17-driven immunity (Contreras et al., 2016), DRD2-signaling has been associated with anti-inflammatory effects (Pacheco et al., 2014). Indeed, a genetic polymorphism of DRD2 gene, which results in decreased receptor expression, has been reported as a risk factor for inflammatory bowel diseases (Magro et al., 2006). Accordingly, although the frequency of Tregs was not changed in the gut, suppressive activity of intestinal Tregs was compromised in inflammatory colitis (Wu et al., 2014), a condition associated to decreased dopamine levels (Magro et al., 2004). Interestingly, the impairment of suppressive Tregs function was abolished by the administration of cabergoline, a DRD2 agonist (Wu et al., 2014). Thus, these results together suggest that, whereas DRD2-signaling in Tregs promotes suppressive function in a healthy gut mucosa containing high dopamine levels, the selective DRD3-signaling in the inflamed gut mucosa containing low dopamine levels favors the inflammatory potential of effector CD4^+^ T-cells promoting further inflammation.

It is noteworthy that one of the main sources of dopamine present in the gut mucosa is given by the commensal gut microbiota. In this regard, it has been described that most dopamine arrives to the gut mucosa as glucuronide conjugated, which is biologically inactive. Nevertheless, *Clostridium* species present in the gut microbiota express β-glucuronidase activity, which catalyzes the production of free dopamine in the gut mucosa (Asano et al., 2012). In addition, recent studies have shown *in vitro* evidence indicating that some components of gut microbiota, including *Bacillus cereaus, Bacillus mycoides, Bacillus subtilis, Proteus vulgaris, Serratia marcescens, S. aureus, E. coli K-12, Morganella morganii, Klebisella pneumoniae and Hafnia alvei*, can also produce dopamine (Clark and Mach, 2016). Of note, a recent study performed with 34 monozygotic twin pairs discordant for MS has shown that when microbiota from the MS twin is transplanted into a transgenic mouse model of spontaneous brain autoimmunity, mice developed autoimmunity with higher incidence than when transplanted with microbiota coming from the healthy twin (Berer et al., 2017). Importantly, when mice were transplanted with microbiota obtained from MS twins, immune cells present in the gut mucosa produced significantly lower levels of the anti-inflammatory cytokine IL-10 than those immune cells of animals receiving microbiota coming from healthy twins. In addition, the analysis in the composition of gut microbiota shows clear differences between healthy and MS twins (Berer et al., 2017). Taken together, these findings indicate that gut microbiota, which can strongly regulate dopamine levels present in the gut mucosa, constitutes a pivotal factor in the control of gut inflammation and in the triggering of MS.

## Author Contributions

RP designed the study and wrote the manuscript. FO-B, CP and FC conducted experiments and acquired data. FO-B, CP, FC and RP analyzed data.

## Conflict of Interest Statement

The authors declare that the research was conducted in the absence of any commercial or financial relationships that could be construed as a potential conflict of interest.

## Abbreviations

Ab: antibody
CFSE: carboxyfluorescein succinimidyl ester
CNS: central nervous system
CD*n*: cluster of differentiation *n*
CFA: complete Freund’s adjuvant
CTLA4: cytotoxic T-lymphocyte antigen 4
DCs: dendritic cells
DR: Dopamine Receptor
DRD*n*: DR D*n*
DRD*n*KO: DRD*n* Knockout
EAE: Experimental Autoimmune Encephalomyelitis
IFN-*n*: interferon *n*
IL-*n*: interleukin *n*
mAb: monoclonal antibody
MFI: mean fluorescence intensity
MOG: myelin oligodendrocyte glicoprotein
OVA: ovalbumin
pOT-II: peptide OVA_323–339_
pMOG: peptide MOG_35–55_
PE: phycoerythrin
PMA: phorbol 12-myristate 13-acetate
RORγt: RAR-related orphan receptor gamma
STAT3: signal transducer and activator of transcription 3
TGF-β: transforming growth factor β
Tregs: regulatory T-cells
Tbet: T-box transcrption factor encoded by TBX21
Th*n*: T helper *n*
WT: wild-type.
