# Supplementary material for: Dopamine Receptor D5 Signaling Plays a Dual Role in Experimental Autoimmune Encephalomyelitis Potentiating Th17-Mediated Immunity and Favoring Suppressive Activity of Regulatory T-Cells
Source: Front Cell Neurosci. 2018 Jul 10;12:192. doi: 10.3389/fncel.2018.00192 (PMC6048279; doi:10.3389/fncel.2018.00192)
Supplement: Supplementary file 1 [file Presentation_1.PDF]

frontiers

Supplementary Material

Dopamine receptor D5 signalling plays a dual role in experimental autoimmune encephalomyelitis potentiating Th17-mediated immunity and favouring suppressive activity of regulatory T-cells

Francisco Osorio-Barrios, Carolina Prado, Francisco Contreras and Rodrigo Pacheco*.

* **Correspondence:** R. Pacheco, Fundación Ciencia & Vida. Avenida Zañartu #1482, Ñuñoa (7780272), Santiago, Chile. Phone: +562 23672046, Fax: +562 22372259, E-mail addresses: rpacheco@cienciavida.org ; rodrigo.pacheco@unab.cl

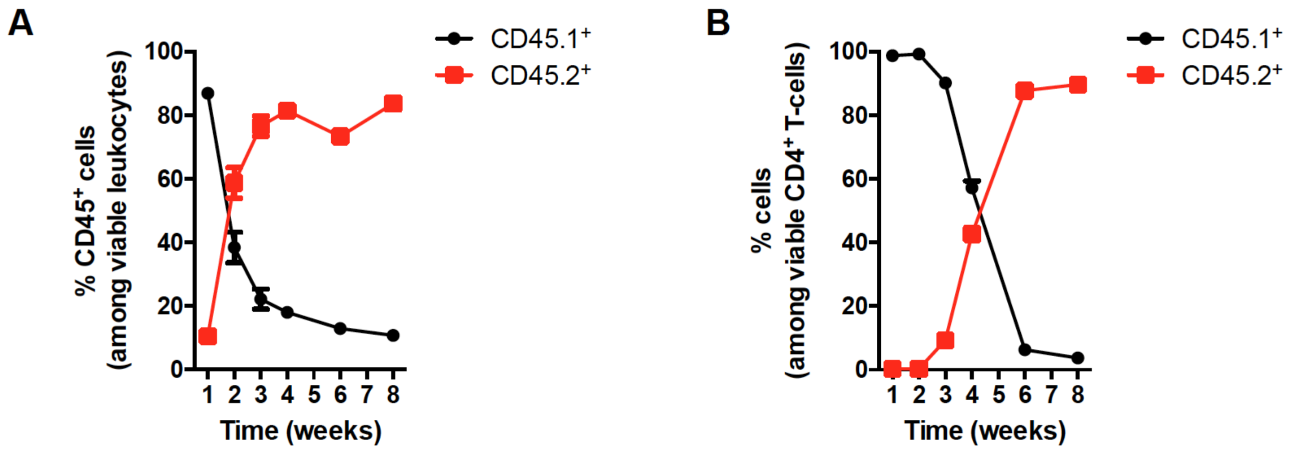

Supplementary Figure 1. Analysis of chimerism after bone marrow transplantation. B6.SJL (*cd45.1*$^{+/+}$) mice were lethally irradiated with two doses of 550 rad each during the same day separated by 3h. One day after irradiation, 10^7 bone marrow cells obtained from C57BL/6 (*cd45.2*$^{+/+}$) were transferred by i.v. route. Degree of chimerism was assessed weekly by tail bleeding. Samples were depleted of red blood cells and then analysed for bone marrow origin either in the entire CD45$^+$ population (**A**) or the CD4$^+$ compartment (**B**). (A and B) The percentage of chimerism corresponds to the percentage of blood cells generated from the donor bone marrow (CD45.2$^+$) with respect to the total blood cells (CD45.1$^+$ plus CD45.2$^+$). Data from a representative result is shown with n = 4 mice. Values are mean ± SEM.

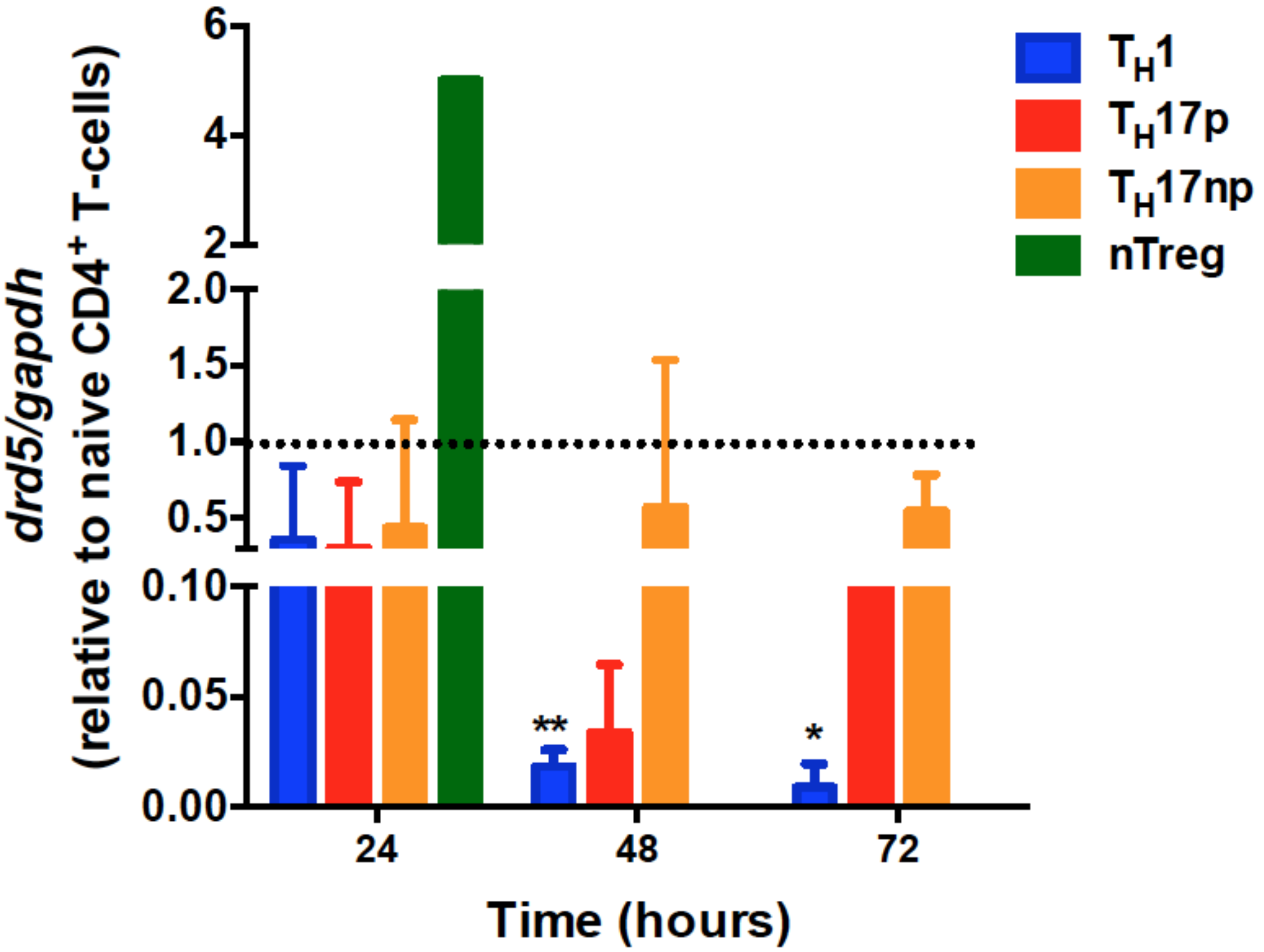

Supplementary Figure 2. Drd5 transcript is differentially expressed in different CD4$^+$ T-cell subsets. Splenic naive (CD4$^+$ CD62L$^+$ CD44$^-$CD25$^-$) T-cell were isolated from WT mice by cell-sorting and immediately analysed or stimulated with anti-CD3 and anti-CD28 Abs in the presence of biased conditions to differentiate to Th1, Th17p or Th17np for 1, 2 or 3 d and then analysed. Splenic CD4$^+$ CD25^{high} T-cells (nTregs) were also isolated from WT mice and immediately analysed. Afterward, total RNA was extracted and *drd5* transcription was analysed by qRT-PCR. *Gapdh* transcription was used as house keeping. Data is represented relative to naive CD4$^+$ T-cells, which correspond to value = 1 (indicated by a dotted line). Values are ± SEM from three independent experiments. *, $p < 0.05$; **, $p < 0.01$ by Student *t*-test.

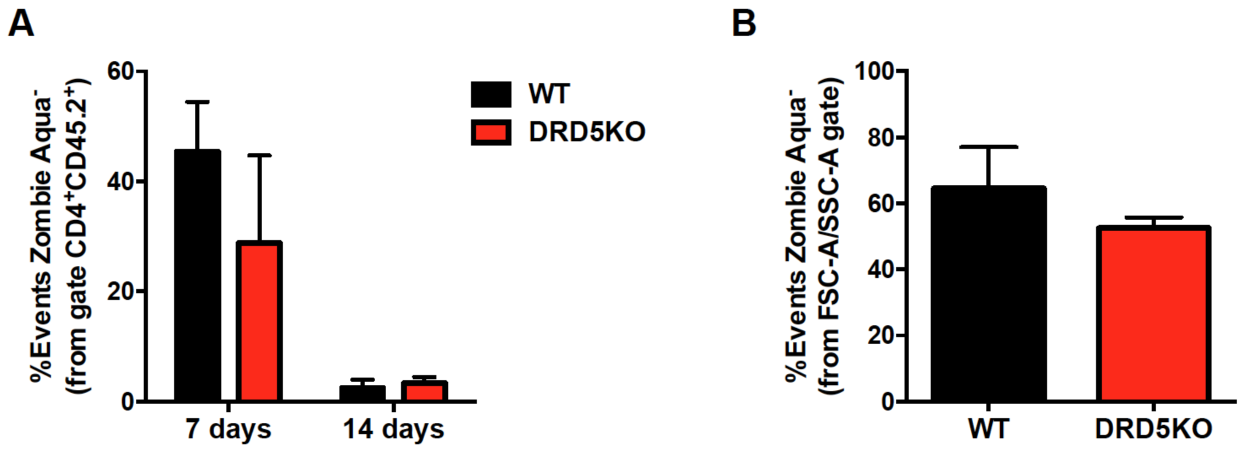

Supplementary Figure 3. **DRD5 deficiency does not affect viability of CD4$^+$ T-cells neither *in vitro* nor *in vivo***. (**A**) Naive CD4$^+$ CD25$^-$ T-cells were isolated from WT/OT-II/*cd45.2*$^{+/+}$ or DRD5KO/OT-II/*cd45.2*$^{+/+}$ mice by cell-sorting and then i.v. transferred (10^5 cells/mouse) into WT/*cd45.1*$^{+/+}$ recipient mice. One-day later, mice were s.c. immunized with 100 µg of pOT-II in CFA and CD4$^+$ T-cells were analysed by flow cytometry in the draining lymph nodes (inguinal lymph nodes) 7 and 14 d after immunization. Quantification of the percentage of living cells (Zombie Aqua$^-$) contained in the CD4$^+$ CD45.2$^+$ population is shown. Values represent mean ± SEM with n = 4 mice per group. No significant differences were detected between genotypes. (**B**) Splenic naive (CD4$^+$ CD62L$^+$ CD44$^-$ CD25$^-$) T-cell were isolated from WT or DRD5KO mice by cell-sorting and immediately stimulated with anti-CD3 and anti-CD28 Abs in the presence of biased conditions to differentiate to Th17np for 5 d and then analysed for viability. Quantification of the percentage of living cells (Zombie Aqua$^-$) contained in the lymphoid population (selected from FSC-A versus SSC-A analyses) is shown. Values represent mean ± SEM from three independent experiments. No significant differences were detected between genotypes.

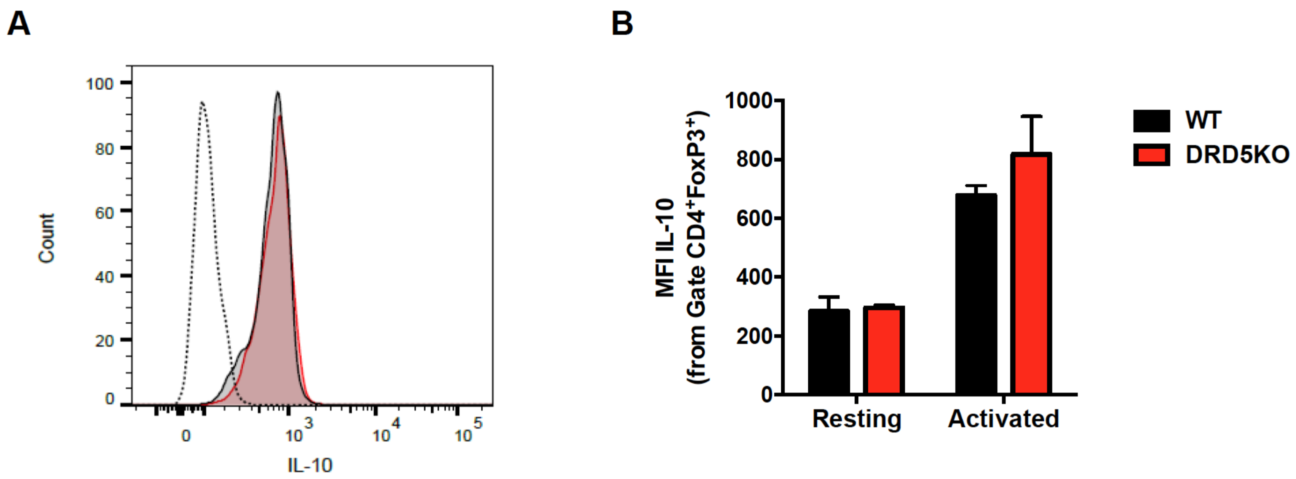

Supplementary Figure 4. DRD5 deficiency in Tregs does not affect the extent of IL-10 production. Splenic $CD4^+$ $CD25^{high}$ T-cells (nTregs) were isolated from WT or DRD5KO mice by cell-sorting and then cultured with IL-2 in the absence (resting) or in the presence (activated) of dynabeads (Treg-to-dynabead ratio 1:2) coated with anti-CD3 and anti-CD28 antibodies for 72 h. Cells were re-stimulated with PMA and ionomycin in the presence of brefeldin A during the last 4 h. Afterwards, cells were immunostained for CD4, then permeabilized and immunostained for Foxp3 and IL-10 and analysed by flow cytometry. (**A**) histograms for IL-10 immunostaining of WT (grey) and DRD5KO (red) activated Tregs in the $CD4^+$ $Foxp3^+$ gate from a representative experiment are shown. Dotted line histogram represent autofluorescence of activated Tregs. (**B**) Quantification of the mean fluorescence intensity (MFI) associated to IL-10 immunostaining in the $CD4^+$ $Foxp3^+$ population is shown. Values represent mean ± SEM from three independent experiments. No significant differences were detected between genotypes.
